# Attack risk for butterflies changes with eyespot number and size

**DOI:** 10.1098/rsos.150614

**Published:** 2016-01-20

**Authors:** Sebastian Ho, Sandra R. Schachat, William H. Piel, Antónia Monteiro

**Affiliations:** 1 Department of Biological Sciences, National University of Singapore, Singapore; 2 Department of Biochemistry, Molecular Biology, Entomology and Plant Pathology, Mississippi State University, Mississippi State, MS 39762, USA; 3 Department of Paleobiology, Smithsonian Institution, PO Box 37012, MRC 121, Washington, DC 20013, USA; 4 Yale-NUS College, Singapore

**Keywords:** paper models, predation, nymphalid, *Mycalesis*, intimidation

## Abstract

Butterfly eyespots are known to function in predator deflection and predator intimidation, but it is still unclear what factors cause eyespots to serve one function over the other. Both functions have been demonstrated in different species that varied in eyespot size, eyespot number and wing size, leaving the contribution of each of these factors to butterfly survival unclear. Here, we study how each of these factors contributes to eyespot function by using paper butterfly models, where each factor is varied in turn, and exposing these models to predation in the field. We find that the presence of multiple, small eyespots results in high predation, whereas single large eyespots (larger than 6 mm in diameter) results in low predation. These data indicate that single large eyespots intimidate predators, whereas multiple small eyespots produce a conspicuous, but non-intimidating signal to predators. We propose that eyespots may gain an intimidation function by increasing in size. Our measurements of eyespot size in 255 nymphalid butterfly species show that large eyespots are relatively rare and occur predominantly on ventral wing surfaces. By mapping eyespot size on the phylogeny of the family Nymphalidae, we show that these large eyespots, with a potential intimidation function, are dispersed throughout multiple nymphalid lineages, indicating that phylogeny is not a strong predictor of eyespot size.

## 1. Introduction

Visual predation is an important selective force shaping the evolution of colour patterning of prey organisms. Anti-predator colour patterns are highly varied and can lead to camouflage and cryptic coloration, conspicuous aposematic coloration, conspicuous patterns that function in either predator deflection or predator intimidation, or any combination of the above [1].

Eyespot patterns are an example of the latter type of protective coloration: a conspicuous circular marking with multiple rings of contrasting colours [2]. Eyespots are most commonly found on the wings of adult Lepidoptera, but can also be found in larvae, and in other insect orders such as Orthoptera, Hemiptera and Coleoptera, as well as in certain fishes, amphibians, reptiles and mammals [2,3]. On the wings of adult Lepidoptera, eyespots vary widely in size and colour, and can be found in different arrangements both on the dorsal and ventral wing surfaces (electronic supplementary material, figure S1).

Butterfly eyespots of the family Nymphalidae appear to have originated once, as a cluster of four or five units on the ventral hindwing in the sister lineage of the subfamily Danainae [4,5]. Eyespots then colonized the forewing and the dorsal surfaces over the course of evolution [6,7].

In the few nymphalid lineages that have been previously studied, eyespots on the dorsal surface can function either in sexual signalling [8,9] or predator deterrence [10–12], whereas those on the ventral surface function in predator deflection [13–15]. These wing surface-specific functions are likely to vary across lineages depending on the basking and courting behaviour of the butterfly.

Two main hypotheses have been investigated regarding the anti-predatory function of eyespots in the Lepidoptera [3,10]. The first is the deflection hypothesis, which posits that small, peripheral eyespots draw the attention of predators to the wing margin, thus deflecting attacks away from vital body parts and towards the edge of the wing [16]. This confers a higher chance of survival because butterflies can survive attacks with only torn wings [16]. Evidence for the deflection hypothesis has been obtained using both vertebrate and invertebrate predators. The presence of eyespots on the margin of the ventral wing surface of *Bicyclus anynana* butterflies did not influence the site of attacks by either lizards (green anole lizard: *Anolis carolinensis*) or adult birds (pied flycatcher; *Ficedula hypoleuca*), but eyespots were found to potentially deflect attacks by naive birds [15,17,18]. Marginal eyespots were found especially effective in deflecting attacks by birds under specific lighting. Blue tits (*Cyanistes caeruleus*) directed their attacks towards the marginal eyespots of *Lopinga achine* butterflies under low light intensity with elevated ultraviolet levels [14]. The deflection hypothesis was further substantiated when invertebrates—namely praying mantids—were used as predators. Wet season *B. anynana* butterflies with conspicuous marginal ventral hindwing eyespots were detected faster by mantids, but had higher survival relative to dry season butterflies with smaller, duller eyespots because the conspicuous marginal eyespots diverted mantids attacks to the wing margins [13].

The second hypothesis is the intimidation hypothesis, which posits that large eyespots deter predation because they produce aversion in predators [3,10]. Numerous studies support this hypothesis. In a series of experiments with passerine birds, eyespots on the wings of live butterflies were shown to deter predation [12,19,20]. When given the choice between real *Junonia almana* wings with eyespots versus wings with the eyespots painted over, great tits (*Parus major*) most frequently attacked the wings that lacked visible eyespots [11]. The intimidation hypothesis is often explained via two non-exclusive mechanisms: the eye-mimicry and the conspicuous-signal mechanisms [3]. In the eye-mimicry mechanism, eyespots are believed to deter predation through their resemblance to the eyes of predators [10]. Studies conducted in the laboratory and in the field with paper models containing eyespots with a centrally located white ‘sparkle’ that mimics light reflecting from the cornea of a three-dimensional vertebrate eyeball were more effective deterrents of predation than eyespots without this feature, or eyespots where the sparkle was rectangular-shaped or positioned in an unnatural position [10,21,22]. In a separate experiment, eyespots that mimicked real owl eyes led to more aversive behaviour from great tits (*P. major*) than eyespots with similar levels of contrast but with reversed colour rings [23]. As for the conspicuous-signal mechanism, eyespots are thought to be intimidating simply owing to their bright, highly contrasting colours. Neophobia and dietary conservatism have been proposed as the behaviours that underlie the effectiveness of eyespots as predation deterrents; that is, eyespots intimidate predators simply owing to the fact that they are salient features [24]. Experiments using artificial prey with eyespots consisting of nested rectangles or triangles of contrasting colours, demonstrate that these salient patterns are as effective as concentric circles in reducing predation risk and demonstrate the importance of conspicuousness in intimidation [25,26].

The eye-mimicry and conspicuousness hypotheses are not mutually exclusive, and it has been argued that a nymphalid eyespot can serve both functions [11,27]. However, more research is needed in order to disentangle the eyespot deflection and intimidation hypotheses, and a preliminary step towards this goal would be to determine a threshold at which an eyespot can be said with certainty to serve at least one of these functions.

As noted above, previous experiments looking into the function of eyespots in interactions with predators have used different butterfly species with a different number of eyespots, of different sizes, present at different locations on the wing, and found that eyespots serve different functions in these different species—either intimidating predators or deflecting attacks towards the wing margin. However, since multiple eyespot characteristics (size, number and position) varied simultaneously in these species, the exact determinant of eyespot function is still not fully understood because no factor was tested in isolation. An exception are the experiments of Stevens *et al.* [25], which used artificial prey (triangles of grey paper) with black-and-white bull's eye eyespots whose distributions varied in size and number. Their results revealed the presence of an intimidation function across the entire range of eyespot sizes tested; however, the smallest eyespots used by Stevens *et al.* (9 mm diameter) were larger in size than those present in species shown to have intimidating eyespots [11,12]. Here, we have conducted a similar study to Stevens' using a single paper prey model that resembled a real nymphalid butterfly, and we manipulated eyespot size, eyespot number or wing size, to test the impact of each of these factors on predation risk. We address the following questions: how does eyespot size affect predation risk? how does eyespot number affect predation risk? and how does wing size alter predation risk of intimidating patterns?

After having addressed this first set of questions, we examined the phylogenetic distribution of the intimidation function in the family Nymphalidae. We measured eyespot size in 255 nymphalid species with a known phylogeny, clustered the eyespots into different size classes and quantified the phylogenetic distribution of each eyespot size class to elucidate the prevalence of different eyespot size classes, such as those tested here, in the natural world.

## 2. Material and methods

### 2.1 Test species and field sites

We conducted field experiments on paper models of the dingy bush brown butterfly, *Mycalesis perseus*, and on six modified pattern variants of this species. *Mycalesis perseus* is a satyrine butterfly that is usually found near shaded areas among tall grasses [28], and is most active during the early morning and late afternoon [29]. This species is relatively common in Singapore, and often occurs at the fringes of nature reserves together with close relatives such as *Mycalesis mineus* and *Mycalesis* *visala* [30]. We conducted each of our predation experiments at three different sites in Singapore where *M. perseus* is naturally found: along North Buona Vista Road (01°29′ N, 103°78′ E; site 1); at View Road (01°27′ N, 103°47′ E; site 2); and along 115 Upper Aljunied Road (01°34′ N, 103°87′ E; site 3) (electronic supplementary material, figure S2).

### 2.2 Preparation of the butterfly models

**Figure 1.**
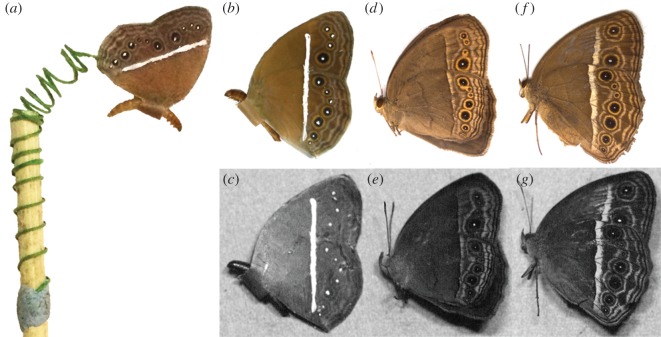
Example of models used in the field study and comparison of visible and UV-reflective patterns of models and natural species. (*a*) Attachment of the butterfly model to the wooden stick. A green metal wire was coiled around the stick, with one end of the wire secured with Blutac. The other end of the wire was glued between the model's two wing surfaces. Photographs captured under visible light (*b*, *d* and *f*) and UV light (*c*, *e* and *g*). (*b*,*c*) Butterfly model of *M. perseus*, which was used in experiment 1. (*d*,*e*) *Mycalesis perseus*, with equal number of eyespots and the transverse band as in (*b*,*c*). (*f*,*g*) *Mycalesis mineus*, a very similar-looking species to *M. perseus*, with a much brighter and thicker band as in (*b*,*c*).

Using a digital photograph of *M. perseus* in its natural resting position [31], we corrected the wingspan and modified the eyespots using Adobe Photoshop CS5.1. A mirror image of the butterfly was created, and a band (approx. 6 mm in length and 5 mm in width) with the same colour as the butterfly's body was added between the two images connecting the mid-section of the body. The butterfly images were then printed in colour on normal paper using a colour laser printer. The paper butterflies were cut out and dipped in paraffin wax to render them waterproof. A live mealworm (*Zophobas morio* larva) was attached to the 6 mm band using a piece of Blutac. A piece of green florist metal wire was coiled around a wooden stick, with one end of the wire secured to the stick using Blutac and the other end glued between the two wings using Scotch Super Glue (figure 1*a*). The metal wire coil, in the field, was extended out from the wooden stick such that the butterfly model swayed in the wind (figure 1*a*). This movement may be important in attracting a predator's attention. Also, in mycalesine butterflies, the white centre of the eyespots and transverse white band reflect ultraviolet light [8] (figure 1*e*,*g*); this too can be important in alluring predatory birds, which have ultraviolet vision. In order to mimic a potentially important factor in facilitating predator approaches, we applied ultraviolet (UV) paint (Fish Vision^TM^ UV Reflective Lure & Jig Paint UV *WHITE*) to the waxed paper butterflies on the white band and in the centre of each eyespot (figure 1*b*). In order to exclude ants and other apterous invertebrate predators, the ends of the wooden sticks were dipped into a pyrethrin solution (ENVIROLE Bed Bug Spray). As a further precaution, the middle section of each stick was coated with approximately 5 cm of deltamethrin and cypermethrin-containing chalk (The LAXMANREKHAA^r^ for cockroaches).

### 2.3 Experimental set-up

We performed six experiments at each of the three study sites. For each experiment, we compared predation of a test model against that of a control model with no eyespots. We used 20 test models and 20 control models per site, totalling 720 model butterflies across all six experiments.

**Figure 2.**
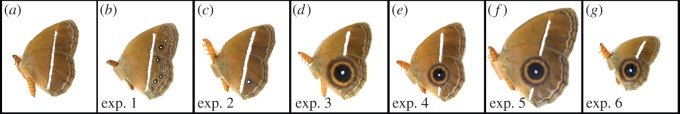
Butterfly models used in the various experiments. (*a*) Non-eyespotted model which was used in every experiment to compare predation with the test pattern. (*b*) Model of *M. perseus*. (*c*) Model with marginal eyespots eliminated except for the Cu1 eyespot. (*d*) Model with a single large eyespot of diameter approximately 8 mm. (*e*) Model with an eyespot obtained by combining the area of all marginal eyespots of the *M. perseus* model. This eyespot has a diameter of approximately 6 mm. (*f*) Model which has enlarged wings and eyespot diameter of approximately 8 mm. (*g*) Model which has reduced wings and eyespot diameter of approximately 6 mm.

The control model for all experiments was produced by covering the eyespots from the *M. perseus* image with surrounding background scales using the Clone Stamp Tool in Adobe Photoshop (figure 2*a*).

In experiment 1, the test pattern was an unmodified image of *M. perseus*with 11 small marginal eyespots: four on the forewing and seven on the hindwing (figure 2*b*). The area of the model's hindwing was approximately 212 mm^2^, which falls within the typical hindwing size of *M. perseus* obtained from the field. The wing area of models was kept constant throughout the study, except in experiments that explicitly tested the effect of wing size.

In experiment 2, all the eyespots of *M. perseus*were eliminated, as in the control pattern, but a Cu1 eyespot on the hindwing was added using a photo taken at higher resolution (to allow for later size manipulation in subsequent experiments) (figure 2*c*). The eyespot used in this experiment had the same diameter as the wild-type eyespot: approximately 2.2 mm, and area of approximately 4.5 mm^2^. The measurement of eyespot diameter included the white centre, black disc and the yellow ring.

In experiment 3, all the eyespots of *M. perseus*were eliminated and replaced with a single eyespot with a diameter of approximately 8 mm (surface area approx. 54 mm^2^) (figure 2*d*). Stevens *et al.* [25] had previously shown that eyespots around this size (9 mm) are capable of intimidating unknown predators in field experiments.

In experiment 4, the individual eyespot featured on the wing had an area that corresponded to the sum of the areas of all eyespots from experiment 1 (surface area approx. 28 mm^2^). The diameter of the resulting eyespot was approximately 6 mm.

For experiments 5 and 6, wing sizes of the models were enlarged and reduced, respectively. Using the model in experiment 4, we expanded the entire butterfly until the eyespot reached 8 mm in diameter (as in experiment 3). This model, used for experiment 5, had a hindwing area of 370 mm^2^ (figure 2*f*). For experiment 6, we shrunk the model in experiment 3 such that the eyespot diameter was reduced from 8 mm to 6 mm. The model's hindwing area in experiment 6 was approximately 115 mm^2^ (figure 2*g*).

At each site, the 40 butterfly models were divided into five sets of eight models each. Each set contained four models of the test pattern and four models of the control non-eyespotted pattern. Within each set, the models were placed in alternate positions, with models spaced approximately 2 m apart (electronic supplementary material, figure S3). Sets were spaced approximately 10 m apart. The butterfly models in each set were placed along a trail, and when trails were not present, the models were placed along a straight line.

After the butterfly models were placed in the field, daily inspections were carried out to score predation events. At each site, models were retrieved when at least half the models of either the control or test patterns had been attacked. This was done in order to prevent rarefaction of models of the preferred type, leaving available for predation only models of the other type. A predation event was scored when the mealworm was partially or fully consumed, or if the entire butterfly went missing. We run the trials from August 2014 to January 2015. The trials were run in the following sequence: experiments 1, 3, 4, 5, 6 and 2. For each of the experiments, we first run the trials at site 1, then site 2 and lastly site 3.

In order to try and determine the identity of the predators, we positioned three camera traps in front of three models in some of the trials.

### 2.4 Statistical analyses

We used Fisher's exact probability test to evaluate differences in predation intensity between model types for each site. We used a paired sample *t*-test, with number of predation events for each type of model in each site as the paired-test variable, to evaluate the effect of the wing pattern treatment across all three sites. A generalized linear mixed model (GLMM) test, with a binomial distribution and logit link function, was performed on the results of experiments 2–6 in order to test whether eyespot size, hindwing size or the interaction between these two factors led to significant differences in predation intensity. The GLMM test can accommodate random effects such as site and the number of days the models were left in the field. All *t*-tests and GLMM test were calculated using IBM SPSS Statistics v. 21.

### 2.5 Phylogenetic distribution of eyespot size

To estimate the phylogenetic distribution of taxa containing eyespots with a likely intimidation function, we measured eyespot area across the family Nymphalidae, based on previous phylogenetic sampling [32]. We examined photographs of the dorsal and ventral wing surfaces of 399 nymphalid species used in previous studies [4,5,7], available at www.lepdata.org. These represent 399 of the approximately 6000 known nymphalid species and 399 of the 540 known nymphalid genera [32]. We identified 255 species with eyespots (as defined in [2]; a roughly circular pattern on the wing, with at least two concentric rings or with a single colour disc and a central pupil), and for each species we made measurements on a single specimen of the sex displaying the largest eyespots. In each specimen, we measured eyespot area in up to 18 different wing sectors distributed across the four wing surfaces: dorsal anterior, dorsal posterior, ventral anterior and ventral posterior (electronic supplementary material, figure S4). The 18 wing sectors selected for measurement were those that contained the largest eyespot (across dorsal and ventral surfaces of the same individual) in at least one of the 255 species examined. Two orthogonal diameters were taken for each eyespot using ImageJ v. 1.48 and the area of the correspondent ellipse was used as a proxy for eyespot area.

We clustered eyespot size data into bins according to *a priori* and *a posteriori* criteria. The *a priori* criterion was the intimidation threshold derived from our experimental results (28.27 mm^2^). We designated a ‘small’ cluster with eyespots below this threshold and a ‘large’ cluster with eyespots above this threshold. For *a posteriori* clustering, we used the NbClust package [33] to identify the optimal number of eyespot-size clusters in our dataset (with maximal between-cluster variance and minimal within-cluster variance) by calculating the Calinski and Harabasz index [34] with the ‘kmeans’ method. We then assigned each measurement to an eyespot-size cluster using the base-R function kmeans [35].

In order to measure the distribution of each eyespot cluster across the phylogeny, we calculated Fritz and Purvis’ *D* [36] using the phylo.d function in the R package caper [37]. Negative values of *D* indicate that a trait has a clumped distribution, meaning that it is strongly associated with particular clades. Values above 1 indicate the opposite situation, in which a trait is dispersed throughout the phylogeny and is not associated with particular clades. Intermediate values between 0 and 1 indicate that a trait is neither highly clumped nor highly dispersed on the phylogeny. We used this metric separately on each wing surface, calculating *D* for all clusters that contained two or more species.

## 3. Results

It took between 1 and 3 days until at least half of the artificial prey from any of the treatment groups were predated upon (electronic supplementary material, table S1). From the total number of predated models across all experiments (392), 24 were completely missing, and the rest had partially or fully consumed mealworms, and often part of the paper as well (electronic supplementary material, figure S5). Non-predated models still had live mealworms after 3 days. The disappearance of the entire butterfly was always accompanied with a directional pull of the wire, either in an upward or downward direction. The upward and downward directional pulls are probably indicative of aerial and ground attacks, respectively. Around 75% of missing models were accompanied with downward directional pulls, while 25% were upward directional pulls. There were no clear biases in the type of model that suffered these attacks.

The camera trap data did not help identify specific predators of the mealworms. This was perhaps because most of the attacks happened relatively fast and the predator was not captured by our motion-activated cameras. In one instance, we were able to photograph the tip of a wing of a flying bird, but could not identify the species, and in no instance did we see evidence of a human intervention.

### 3.1 Field experiments

**Figure 3.**
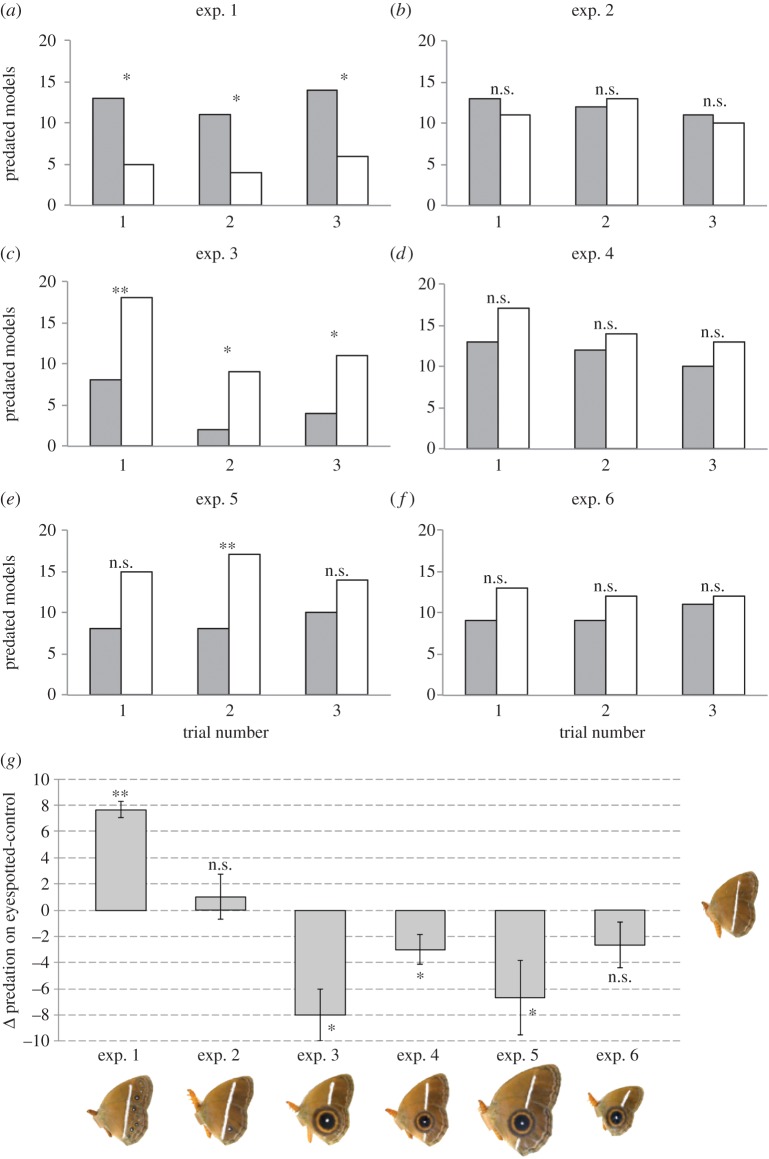
Predation results for each of the six experiments. (*a*–*f*) Number of predated eyespotted models in each site are depicted in grey bars, and non-eyespotted models in white bars. (*g*) Mean differences in the number of predation events between the test pattern and non-eyespotted pattern for each of the six experiments. Error bars correspond to 95% confidence intervals (CIs) across the three sites.*p<0.05; **p<0.01; n.s., not significant.

Experiment 1 (exp. 1) showed that models with multiple small marginal eyespots are attacked significantly more than non-eyespotted models (figure 3*a*,*g*; electronic supplementary material, table S1). Experiment 2 (exp. 2) showed that butterfly models with just one marginal eyespot suffer similar predation relative to models without any eyespots (figure 3*b*,*g*; electronic supplementary material, table S1). Experiment 3 (exp. 3) showed that models with a single, large eyespot of approximately 8 mm in diameter suffered significantly less predation than non-eyespotted models (figure 3*c*,*g*; electronic supplementary material, table S1). For all three experiments, we recovered similar results within and across sites.

Experiment 4 (exp. 4) showed that models with a single large eyespot, with the same total surface area as the multiple, small eyespots in *M. perseus*, also suffered less predation than non-eyespotted models, despite the eyespots being somewhat smaller (6 mm diameter) than the eyespots used in exp. 3. These differences were not significant for any of the individual sites (figure 3*d*), but were significant across all three sites (figure 3*g*; electronic supplementary material, table S1). This experiment showed that size of individual eyespots, not total eyespot surface area, determines intimidation.

By comparing the results of exp. 2 with those of exp. 1 or with those of exps. 3 and 4, we can ask how changes in signal intensity, via increases in signal number (exp. 1), or increases in signal size (exps. 3 and 4) affected predation risk. We found that an increase in signal number significantly increased predation risk (paired *t*-test; *t*_2_=12.124, *p*=0.007), whereas an increase in signal size significantly decreased predation risk. This effect was significant for the 8 mm eyespot treatment (paired *t*-test; *t*_2_=4.914, *p*=0.039), but not for the 6 mm eyespot treatment (paired *t*-test; *t*_2_=2.524, *p*=0.128).

Experiments 5 (exp. 5) and 6 (exp. 6) tested whether variation in wing size alters the eyespot intimidation effect. In exp. 5, we used an 8 mm eyespot, as in exp. 3, but arrived at this eyespot size by increasing the wing size of the model used in exp. 4. Despite a single site showing these differences as significant (figure 3*e*), the non-eyespotted models suffered higher predation than the eyespotted models across all sites, as in exp. 3 (figure 3*g*; electronic supplementary material, table S1). In exp. 6, we used a 6 mm eyespot, as in exp. 4, but arrived there by reducing the wing size of the model used in exp. 3. As in exp. 4, non-eyespotted models suffered a higher (albeit non-significant) number of attacks relative to eyespotted models (figure 3*f*), and attack frequencies closely followed those of exp. 4. However, unlike exp. 4, the difference remained insignificant across the three sites (figure 3*g*; electronic supplementary material, table S1; *p*=0.094). In order to compare the results of exps. 3 and 5, and those of exps. 4 and 6—where eyespot sizes were constant but wing sizes were different—we performed a paired *t*-test on the predation differences between non-eyespotted and eyespotted models between the two experiments. This test showed no significant differences in outcome for exps 3 and 5 (*t*=0.8; *p*=0.508) or for exps 4 and 6 (*t*=0.378, *p*=0.742), indicating that wing size does not significantly alter predation intensity of models with similarly sized eyespots, given that the eyespots are above 6 mm in diameter.

To examine the effect of eyespot size, wing size and the interaction between these two factors on predation intensity, we used a GLMM with predation data from exps. 2–6. We found a non-significant interaction effect of eyespot size and wing size (AICc = 2,632; *F*=1.475, *p*=0.225). By running another GLMM without this term, we decreased the Akaike information criteria (AICc) values, increased the model's fit to the data, and found that eyespot size, but not wing size, had a highly significant effect on predation intensity (table 1). Because the eyespots in exp 2 were much smaller than the eyespots in exps. 3–6, we run a similar analysis to the one above but using only data from exps. 3–6. We obtained the same results as in the previous analysis (table 1). The interaction effect was non-significant (*F*=0.938; *p*=0.333; AICc = 2127), and eyespot size, but not wing size, was found to explain most of the predation variation in our data (table 1).

**Table 1.** Results of GLMM analysis on factors affecting predation probability across exps. 2–6 and 3–6. (We used a binomial distribution (1 versus 0 for each model), a logit link function, and total eyespot size and hindwing size as fixed factors.)

					95% CI		
exps.	AICc	fixed effects	*F*-values	coefficients	lower	upper	*p*
2–6	2615	total eyespot size	37.112	0.026	0.018	0.035	<0.0001
		hindwing size	1.957	−0.002	−0.005	0.001	0.162
3–6	2110	total eyespot size	40.458	0.030	0.021	0.039	<0.0001
		hindwing size	2.591	−0.003	−0.006	0.001	0.108

In summary, the experiments above revealed that many small, marginal eyespots led to a high level of predation, whereas single large eyespots greater than 6 mm in diameter intimidated predators. Neither the presence of a single small eyespot (relative to no eyespots) nor changes in wing size had a significant effect on predation intensity (figure 3*g*).

### 3.2 Nymphalid diversity and phylogeny

**Figure 4.**
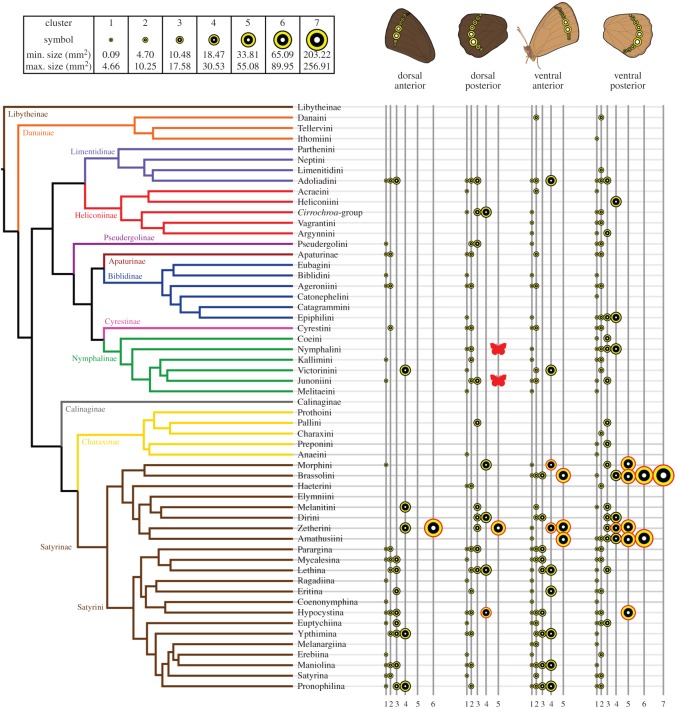
Phylogenetic distribution of eyespot size in Nymphalid butterflies. We mapped the *a posteriori* eyespot-size clusters (depicted by eyespot symbols of different sizes on the right) rather than individual eyespot sizes. Eyespots larger than 6 mm in diameter are highlighted in red. The two red butterfly icons denote *Aglais io* and *Junonia almana*, from the tribes Nymphalini and Junoniini, respectively, whose eyespot patterns are known to intimidate vertebrate predators [11,12].

Our study shows that single eyespots of 6 mm in diameter or greater have an intimidation effect on predators. Assuming that this intimidation function will be present in other species with at least one eyespot of this size, regardless of wing size, we explored the phylogenetic distribution of large eyespots by mapping the distribution of eyespot-size clusters onto a phylogeny of nymphalid subtribes (for the ultra-diverse Satyrini) and tribes (for all other lineages) (figure 4). We also calculated Fritz and Purvis' *D* for each eyespot-size cluster in order to quantify the distribution of likely intimidating eyespots on the nymphalid phylogeny.

Eyespots over 6 mm in diameter are predominantly present in the subfamily Satyrinae, which accounts for 42.61% (170 out of 399) of the species sampled (figure 4). A total of 86.36% (19 out of 22) of the eyespots that surpass the 6 mm intimidation threshold occur on the ventral surface, with the remaining 13.64% (3 out of 22) on the dorsal surface. Large dorsal eyespots occur in species that also have large (greater than 6 mm) eyespots in the homologous wing sector on the ventral surface: *Zethera incerta*, *Neorina crishna* and *Tisiphone abeona* (electronic supplementary material, table S2).

We recovered seven *a posteriori* eyespot-size clusters, which we ranked according to eyespot size. On all four of the wing surfaces, eyespots become increasingly rare as their size increased (table 2; electronic supplementary material, figure S6). Our calculations of *D* for the *a posteriori* eyespot-size clusters show that phylogeny does not predict distribution of eyespot size. Nearly all eyespot-size clusters yielded values of *D* between 0 and 1, indicating that eyespot size is neither strongly clumped nor strongly dispersed across the phylogeny. On all wing surfaces, cluster 4 (surface area 18.47–30.53 mm^2^) yielded the highest value of *D*, indicative of wide phylogenetic dispersion; this is the only *a posteriori* cluster for which we recovered values above 1. This cluster includes eyespots with a diameter of 6 mm, which were experimentally shown to intimidate predators. The only eyespot cluster that yielded a negative value of *D*, indicating strong phylogenetic signal, is cluster 7, which contains the largest eyespots and includes only two species.

**Table 2.** Composition of the *a priori* and *a posteriori* eyespot-size clusters used in this study. (Table depicts, minimum and maximum surface area (in mm^2^) of eyespot sizes contained in each cluster; the number of species (no. spp.) represented in that cluster broken down by wing surface; and *D* containing a measure of phylogenetic dispersion of eyespots of that size.)

				wing surface							
				dorsal anterior		dorsal posterior		ventral anterior		ventral posterior	
criterion	cluster	min. size	max. size	no. spp.	*D*	no. spp.	*D*	no. spp.	*D*	no. spp.	*D*
*a posteriori*	1	0.09	4.66	26	0.95	44	0.52	74	0.59	109	0.66
	2	4.70	10.25	20	0.46	19	0.98	35	0.52	42	0.60
	3	10.48	17.58	13	0.51	10	0.80	13	0.76	19	0.47
	4	18.47	30.53	5	1.09	5	1.53	9	1.23	9	0.82
	5	33.81	55.08	0		1		3	0.70	8	0.58
	6	65.09	89.95	1		0		0		3	0.74
	7	203.22	256.91	0		0		0		2	−0.36
*a priori*	small	0.09	26.77	64	0.20	77	0.54	129	0.41	178	0.57
	large	28.97	256.91	1		2	1.71	5	0.25	14	−0.17

Our *a priori* clusters show a similar lack of phylogenetic signal (table 2; electronic supplementary material, figure S6). Most values of *D* fall between 0 and 1, phylogenetic overdispersion is rare and associated only with large eyespots, and the only negative value of *D*—indicating phylogenetic clumping—corresponds to the largest eyespots on the ventral hindwing. Our phylogenetic analyses therefore show that phylogeny is not a strong predictor of eyespot size and potential intimidation function.

## 4. Discussion

Here, we show that multiple small (approx. 2.2 mm in diameter) marginal eyespots attract predators, whereas single large (greater than 6 mm) eyespots intimidate predators. Single small marginal eyespots neither attract nor intimidate predators. Unlike the experiments of Stevens *et al.* [25], we did not observe that ‘small and many’ and ‘few and large’ eyespot patterns were equally effective in predator intimidation. Their experiments showed that three ‘small’ 9 mm eyespots were as effective in intimidating predators as one larger 15 mm eyespot with the same total area. The reason for this discrepancy is probably because Stevens and colleagues worked with paper models where the smallest eyespots were greater than 9 mm in diameter, and, thus, intimidating on their own according to our findings. Our experiments show that paper models with 11 small eyespots actually draw predators towards the models, whereas models with a single eyespot with the same total area intimidate predators. Our small eyespots are similar in size to the most prevalent eyespots across the family Nymphalidae. The absolute size of an individual eyespot thus contributes to attack risk of our model butterflies.

The reason why models with multiple, small eyespots drew more attacks relative to non-eyespotted models is likely to be related to the higher conspicuousness of these models. Previous work demonstrated that small marginal eyespots could be effective in deflecting the attacks of birds and mantids [13,15,17,18,38,39]. Although we did not directly observe the deflective function in our paper models, our results are consistent with the idea behind the deflection hypothesis—small, conspicuous, marginal eyespots may attract more attention but ultimately increase prey fitness by increasing the likelihood of escape [13,40]. Associated with this function, however, is the prediction that attack marks should have been visible on the wing margin of the attacked models. Most wings, however, displayed no such marks. A possible explanation is that attacks were indeed directed to the eyespots but did not leave a mark on the robust, waxed, paper wings. Alternatively, the attachment of the models to coiled metal wires meant that any possible attacks directed from behind at the eyespots caused the models to move forward in the same direction, minimizing damage to the models. One additional possibility is that, since these model butterflies are incapable of exhibiting any form of behaviour such as wing flicking, predators may direct their strike to the moving live mealworm at close range instead of attacking the immobile wings.

Our experiments do not provide a definite eyespot size threshold for the transition into an intimidating function. This threshold is likely to vary among different predators, and probably depends on other factors such as eyespot number, additional wing pattern elements, wing colours, butterfly behaviour such as wing flicking and stridulation [12], and properties of the background habitat. Previous experiments showed that different predators have unique responses to eyespots, implying that each predator community exerts its own selective pressures [11–15,17–20,41]. However, our field experiments have assessed eyespot effectiveness in a diverse, natural predator community instead of laboratory conditions involving a single predator [27,41].

Our camera trap data did not help identify likely predators. It is possible that some of the attacks were produced by insects, such as crickets and grasshoppers, and others by nocturnal animals using non-visual cues. It is unclear whether these predators predate live butterflies or have shaped the evolution of their wing patterns. However, the results presented above document significant and repeatable differences in predation risk across the treatment groups, which cannot easily be explained by accidental removal of models by humans, by wind or by non-visual nocturnal predators.

Unlike manipulations of eyespot size and eyespot number, our manipulations of wing size showed that this parameter did not significantly affect predation intensity. Vallin *et al.* [20] similarly reported that peacock butterflies, though smaller than hawkmoths, fared better when presented to blue tits and great tits. Interestingly, both species had similar eyespot sizes, and the enhanced survival of the smaller of the two was primarily attributed to its typical wing flicking behaviour following detection, which increased the intimidation effect of eyespots.

Our upward manipulation of eyespot size resulted in a decreased length of the transverse white band, which may have reduced overall model conspicuousness. It is therefore possible that the reduced attacks observed for models with enlarged eyespots could be owing to the reduced size of the white band in these models rather than to their larger eyespots. Comparisons among the enlarged eyespot groups, however, suggest that this is not the case. In particular, the length of the white band is greater in models from exp. 5 relative to models in exp. 4, yet models in exp. 5 suffered less predation. In addition, models from exp. 3 and those for exp. 6 have a similar size of the white band yet differ considerably in attack risk owing to the size of their eyespots. Future experiments with eyespot-size manipulated paper models should perhaps dispense with the transversal banding pattern altogether to avoid the inevitable unintentional alterations to this pattern, as a result of the eyespot size manipulations.

Our phylogenetic investigation and previous experimental work indicate that likely intimidating eyespots (those larger than 6 mm) are present in two nymphalid subfamilies, the Nymphalinae and the Satyrinae, and are dispersed throughout the phylogenies of both subfamilies. In addition to the 255 species measured for this study, two additional species, *Aglais io* and *Junonia almana*, from the nymphaline tribes Nymphalini and Junoniini, respectively, are known to have wing patterns that intimidate birds [11,12]. The ‘eyespot-like’ wing pattern on the dorsal forewing of *A. io*, made up of multiple fused marginal eyespots and more proximal bands of colour, measures approximately 7.5 mm in diameter. The largest dorsal posterior eyespot of *J. almana* measures approximately 6.5 mm in diameter. More complete species-level sampling, including multiple species per genus, is likely to reveal additional eyespots that surpass a diameter of 6 mm and give a more complete picture of where the intimidation function of eyespots may have additionally evolved. At present, nymphalid eyespots larger than 6 mm are known only from the subfamilies Nymphalinae and Satyrinae. These subfamilies are very distantly related [32] (figure 4), an observation that highlights the wide phylogenetic dispersion of large, and possibly intimidating eyespots.

One important point to keep in mind, however, is that the 6 mm intimidation size threshold discovered here pertains to the specific community present in grasslands bordering forests in Singapore. Given that the same sized eyespot may be intimidating for smaller predators of a community, and non-intimidating or deflective for larger predators [27,42], it is likely that once predator composition varies, the size threshold will also vary. Only comparative work across multiple habitats and communities, using constant sized prey models, will be able to establish how large eyespots need to be to become intimidating in those communities. A wing pattern with one single large eyespot—suggested by our experiments to be intimidating to predators—is not particularly common in nature, but does occur, for example, in *Faunula leucoglene* (Nymphalidae: Satyrinae: Amathusiini). Future work on the effects of single, large eyespots should focus on the natural habitats of butterflies with this eyespot pattern.

A recent study showed increased effectiveness of intimidating eyespots when they occur in pairs [43]. In contrast to previous findings [44], the authors of this study suggested that intimidating eyespots are most likely to evolve on the dorsal surface. They propose that since eyespots on these dorsal surfaces are visible simultaneously when the wings are open, a pair of simultaneously visible intimidating eyespots will evolve more easily on the dorsal surface than on the ventral surface. However, this suggestion is contradicted by the results of our size survey across nymphalids, in which eyespots larger than 6 mm in diameter were primarily found on the ventral surface. Furthermore, species that have one or more dorsal eyespots larger than 6 mm in diameter often have multiple such eyespots on the ventral surface (electronic supplementary material, table S3). The evolution of multiple ventral eyespots, including large eyespots, therefore does not appear to be rare. Therefore, intimidation function could be conferred by pairedness on the ventral surface as well as the dorsal surface.

Different studies have suggested various determinants of eyespot intimidation function: the sum of total eyespot area [25]; eyespot pairedness, regardless of size [43]; and in this study, individual eyespot size. To further explore how these variables interact, future experimental work could include additional iterations of the models tested here, varying in eyespot number as well as size. Future comparative work could also investigate how eyespot number and size vary among natural species.

Eyespots evolved first on the ventral hindwing, around 90 Ma [4,5]. Our mapping of eyespot size on the nymphalid phylogeny shows that large eyespots (above 6 mm) and with a likely intimidation function are present in only a few derived lineages. This suggests that the eyespots' original function may have been in predator deflection, with the intimidation function evolving sporadically, as a derived trait. Large, intimidating eyespots are most common in the locations on the ventral hindwing where eyespots first appeared 90 Ma. A more detailed examination of the evolution of nymphalid eyespot function will require sampling of eyespot size in all wing locations and would also benefit from denser sampling at the species level.

## Supplementary Material

Supp. Figure 1. Eyespot size and number diversity in the Nymphalidae. Eyespots of various sizes can be found on different wing sectors on both the dorsal and ventral surfaces. (a) Dorsal view of Junonia almana, (b) Dorsal view of Proterebia afra, (c) Dorsal view of Algais io, (d) Ventral view of Caligo telamonius, (e) Ventral view of Mycalesis terminus and (f) Ventral view of Ypthima baldus.

## Supplementary Material

Supp. Figure 2. Field sites used in this study. General terrain of field site at (a) North Buona Vista Road (Trial 1), (b) View Road (Trial 2), (c) 115 Upper Aljunied Road (Trial 3). (d) A map of Singapore indicating the three study localities with red circles.

## Supplementary Material

Suppl. Figure 3. Diagrammatic placement of a set of eight models in each trial. Four of these sets were placed 10 m apart at each field site. Four models of a test eyespotted pattern and four models of the control non-eyespotted pattern were placed in more or less a straight fashion in alternating positions, with each model approximately 2 meters apart from each other.

## Supplementary Material

Suppl. Figure 4. The eyespots measured for the phylogenetic component of the study. Each of these eyespots is the largest on its respective wing surface in at least one of the 255 nymphalid species identified with eyespots. Measured eyespots are outlined in red; those not measured are outlined in black.

## Supplementary Material

Suppl. Figure 5: A few examples of the models that suffered predation. These models were still attached to the green wire but the mealwom was missing and the paper was chewed up to variable degrees. Sometimes the complete paper model was missing.

## Supplementary Material

Suppl. Figure 6. An illustration of the eyespot cluster composition outlined in Table 2. The figure depicts the minimum and maximum eyespot size in each cluster, and the number of species whose largest eyespot falls into each cluster (separated by wing surface).

## Supplementary Material

Suppl. Table 1: Number of eyespotted and non-eyespotted models predated in each trial for the 6 experiments. Number of days that models were left in the field, and the t and p values (two-tailed) obtained from paired sample t-tests on each experiment. Means and standard deviations (SD) for each experiment are also reported.

## Supplementary Material

Suppl. Table 2. Occurrence of eyespots that surpass the intimidation size threshold discovered in this study. These eyespots have a surface area 28.27 mm2 or larger.

## Supplementary Material

Suppl. Table 3: Eyespot size for species in which at least one eyespot has a surface area 28.27 mm2 or larger. For each species we measured eyespot surface area in up to 18 different wing sectors (Suppl. Fig. 3). The 18 wing sectors selected for measurement were those that contained the largest eyespot in at least one of the 255 species examined.

## Data accessibility

Eyespot size data across nymphalids is available from Dryad: .
