# Supplementary figures and images for: Attack risk for butterflies changes with eyespot number and size
Source: R Soc Open Sci. 2016 Jan 20;3(1):150614. doi: 10.1098/rsos.150614 (PMC4736945; doi:10.1098/rsos.150614)

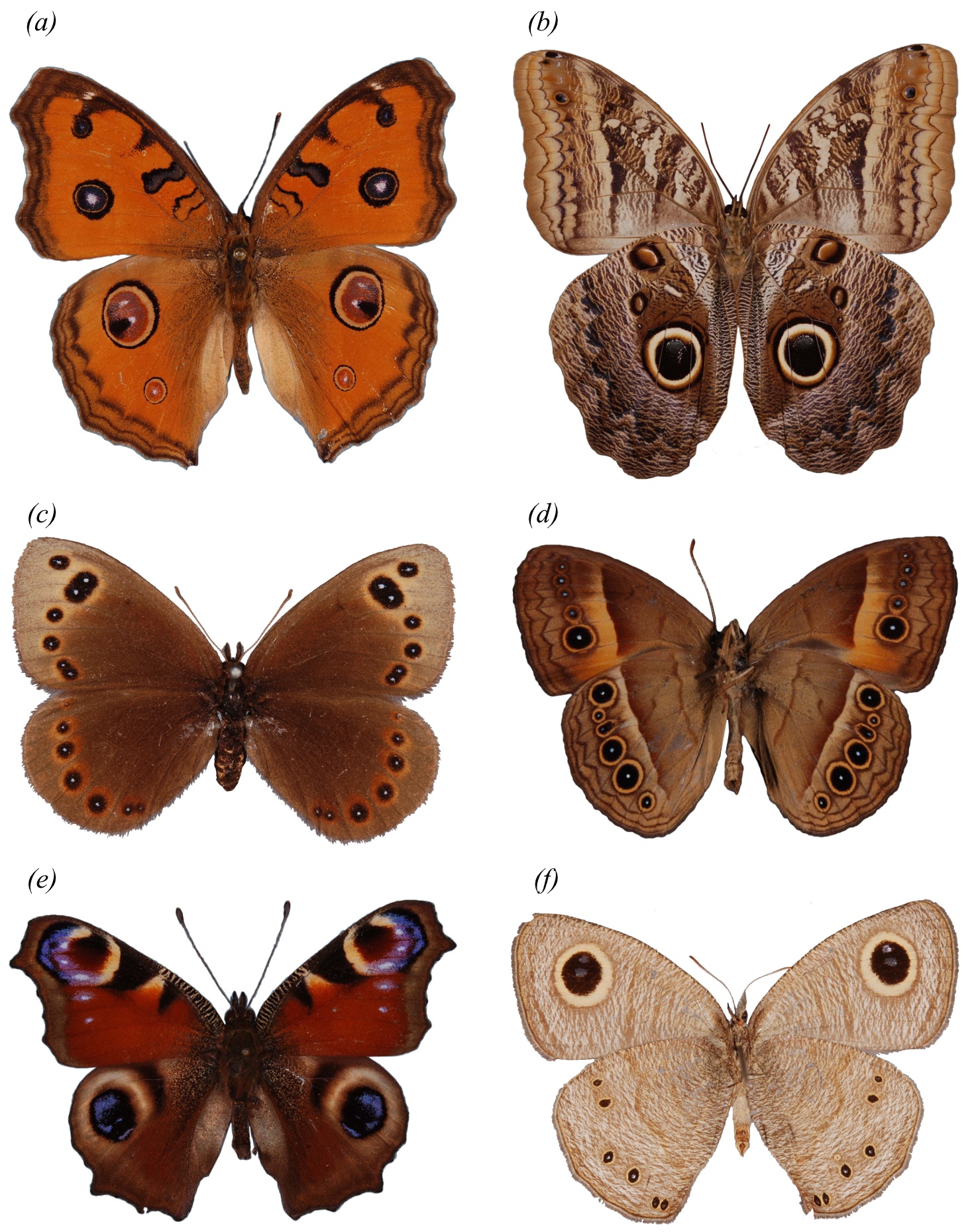

Supplement: Supp. Figure 1. Eyespot size and number diversity in the Nymphalidae. Eyespots of various sizes can be found on different wing sectors on both the dorsal and ventral surfaces. (a) Dorsal view of Junonia almana, (b) Dorsal view of Proterebia afra, (c) Dorsal view of Algais io, (d) Ventral view of Cal [file rsos150614supp1.jpg]

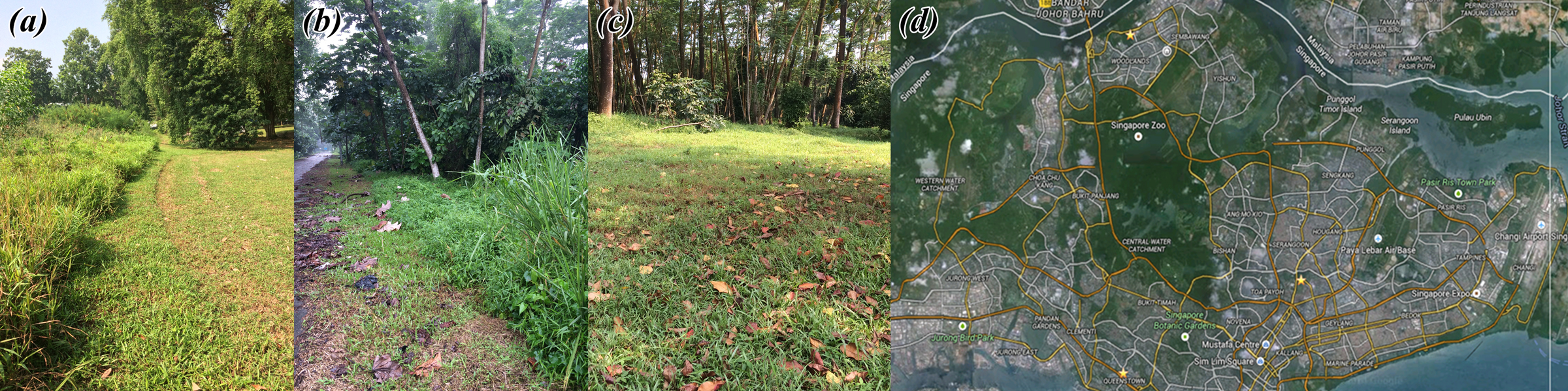

Supplement: Supp. Figure 2. Field sites used in this study. General terrain of field site at (a) North Buona Vista Road (Trial 1), (b) View Road (Trial 2), (c) 115 Upper Aljunied Road (Trial 3). (d) A map of Singapore indicating the three study localities with red circles. [file rsos150614supp2.jpg]

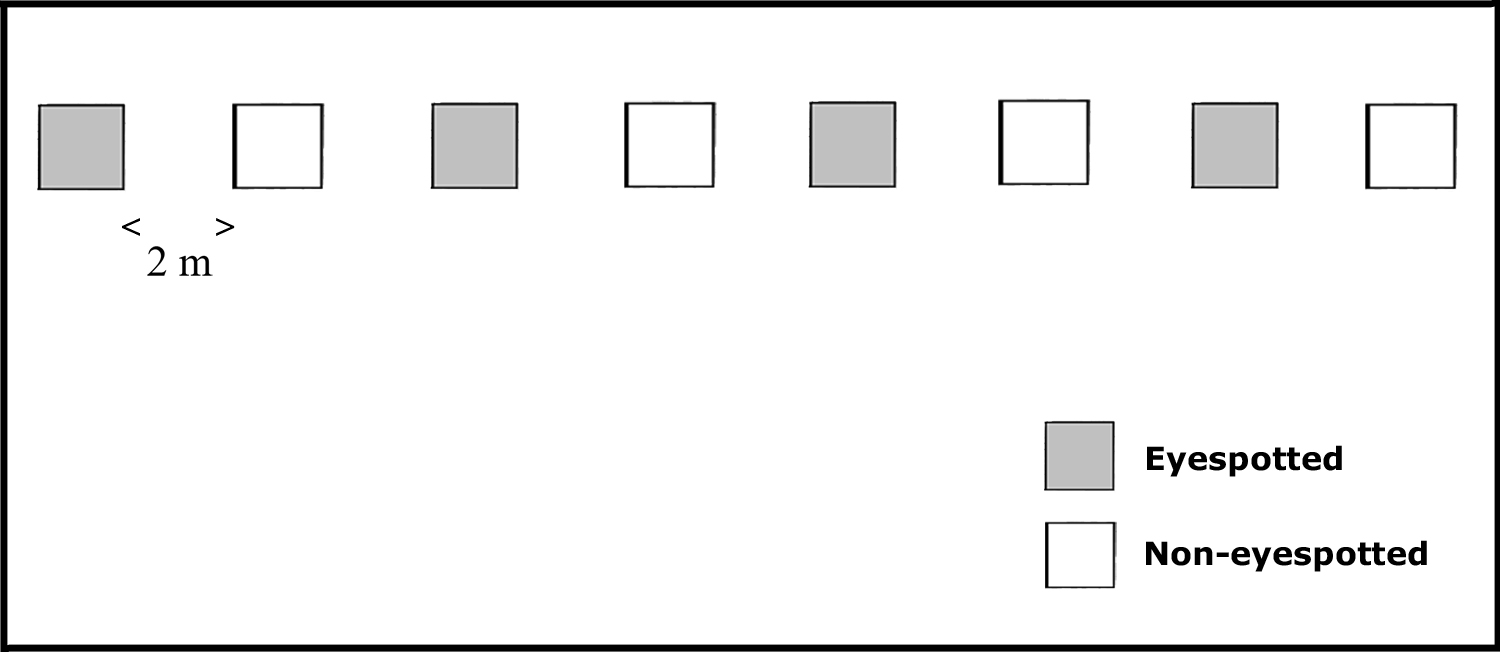

Supplement: Suppl. Figure 3. Diagrammatic placement of a set of eight models in each trial. Four of these sets were placed 10 m apart at each field site. Four models of a test eyespotted pattern and four models of the control non-eyespotted pattern were placed in more or less a straight fashion in alternating p [file rsos150614supp3.jpg]

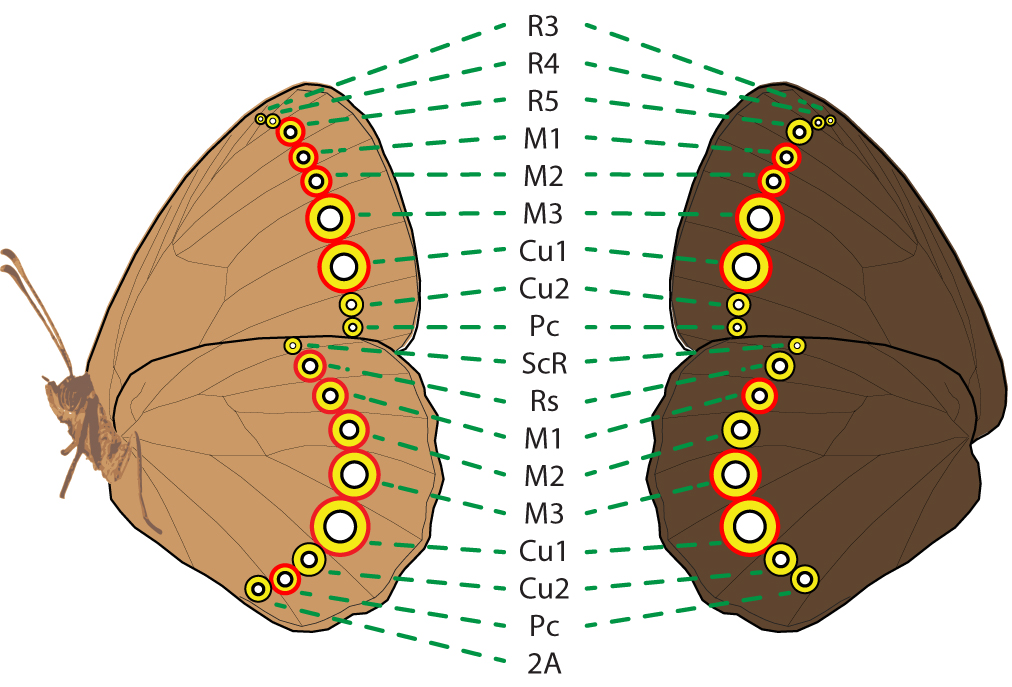

Supplement: Suppl. Figure 4. The eyespots measured for the phylogenetic component of the study. Each of these eyespots is the largest on its respective wing surface in at least one of the 255 nymphalid species identified with eyespots. Measured eyespots are outlined in red; those not measured are outlined in bl [file rsos150614supp4.jpg]

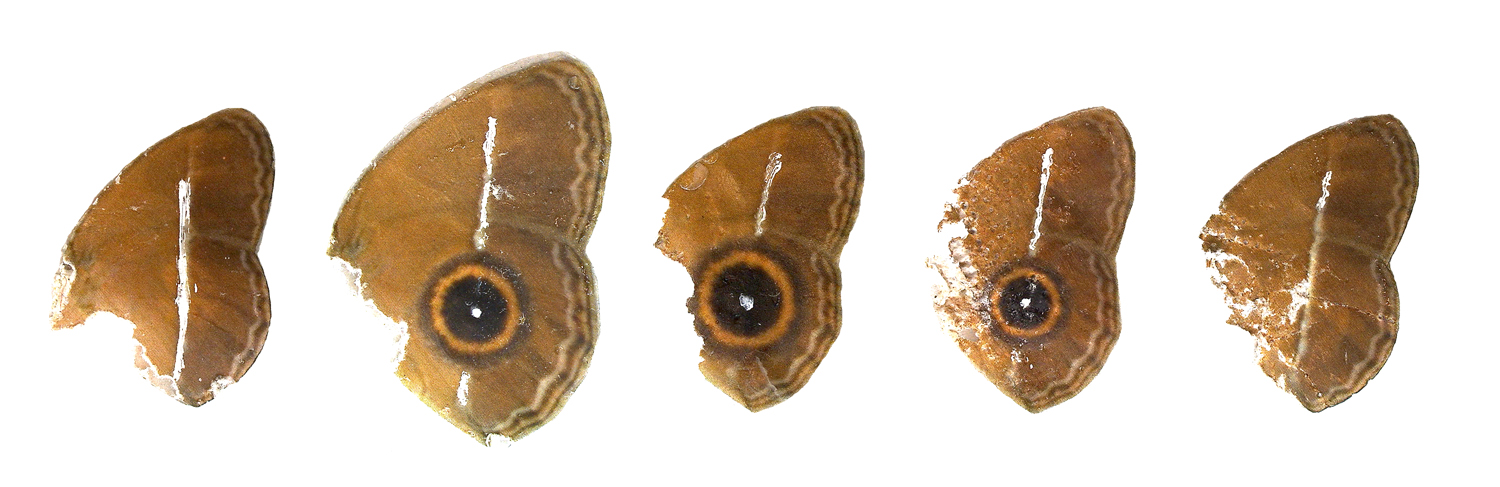

Supplement: Suppl. Figure 5: A few examples of the models that suffered predation. These models were still attached to the green wire but the mealwom was missing and the paper was chewed up to variable degrees. Sometimes the complete paper model was missing. [file rsos150614supp5.jpg]

Eyespot Size

Number of Species in Cluster

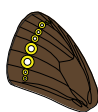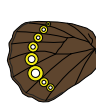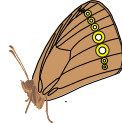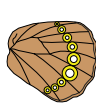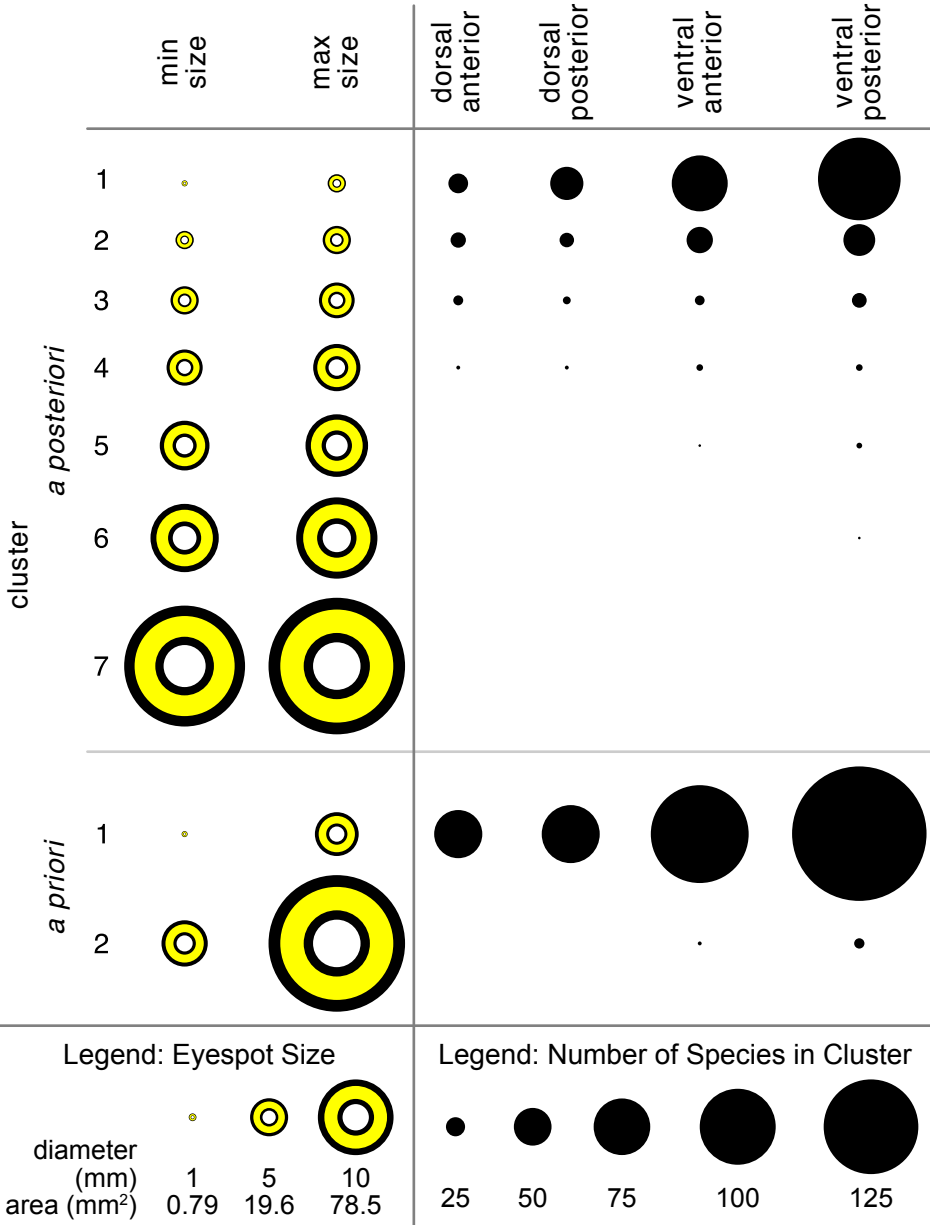

Supplement: Suppl. Figure 6. An illustration of the eyespot cluster composition outlined in Table 2. The figure depicts the minimum and maximum eyespot size in each cluster, and the number of species whose largest eyespot falls into each cluster (separated by wing surface). [file rsos150614supp6.pdf]
