# Supplementary material for: Attack risk for butterflies changes with eyespot number and size
Source: R Soc Open Sci. 2016 Jan 20;3(1):150614. doi: 10.1098/rsos.150614 (PMC4736945; doi:10.1098/rsos.150614)
Supplement: Suppl. Table 1: Number of eyespotted and non-eyespotted models predated in each trial for the 6 experiments. Number of days that models were left in the field, and the t and p values (two-tailed) obtained from paired sample t-tests on each experiment. Means and standard deviations (SD) for each expe [file rsos150614supp7.docx]

**Suppl. Table 1: Number of eyespotted and non-eyespotted models predated in each trial for the 6 experiments**. Number of days that models were left in the field, and the t and *p* values (two-tailed) obtained from paired sample t-tests on each experiment. Means and standard deviations (SD) for each experiment are also reported.

| Experiment | Trial | Number of days in field | Number of eyespotted models predated | Number of  non-eyespotted models predated | t (df = 2) | *p* |
| --- | --- | --- | --- | --- | --- | --- |
| 1 | 1 | 3 | 13 | 5 | 23 | 0.002 |
|  | 2 | 3 | 11 | 4 |  |  |
|  | 3 | 1 | 14 | 6 |  |  |
| Mean |  |  | 12.7 | 5 |  |  |
| SD |  |  | 1.5 | 1 |  |  |
| 2 | 1 | 1 | 13 | 11 | 0.756 | 0.529 |
|  | 2 | 2 | 12 | 13 |  |  |
|  | 3 | 2 | 11 | 10 |  |  |
| Mean |  |  | 12 | 11.3 |  |  |
| SD |  |  | 1 | 1.5 |  |  |
| 3 | 1 | 3 | 8 | 18 | -8 | 0.015 |
|  | 2 | 1 | 2 | 9 |  |  |
|  | 3 | 2 | 4 | 11 |  |  |
| Mean |  |  | 4.7 | 12.7 |  |  |
| SD |  |  | 3.1 | 4.7 |  |  |
| 4 | 1 | 2 | 13 | 17 | -5.196 | 0.035 |
|  | 2 | 1 | 12 | 14 |  |  |
|  | 3 | 2 | 10 | 13 |  |  |
| Mean |  |  | 11.7 | 14.7 |  |  |
| SD |  |  | 1.5 | 2.1 |  |  |
| 5 | 1 | 1 | 8 | 15 | -4.588 | 0.044 |
|  | 2 | 2 | 8 | 17 |  |  |
|  | 3 | 1 | 10 | 14 |  |  |
| Mean |  |  | 8.7 | 15.3 |  |  |
| SD |  |  | 1.2 | 1.5 |  |  |
| 6 | 1 | 2 | 9 | 13 | -3.024 | 0.094 |
|  | 2 | 2 | 9 | 12 |  |  |
|  | 3 | 1 | 11 | 12 |  |  |
| Mean |  |  | 9.7 | 12.3 |  |  |
| SD |  |  | 1.2 | 0.6 |  |  |
