# Supplementary material for: Attack risk for butterflies changes with eyespot number and size
Source: R Soc Open Sci. 2016 Jan 20;3(1):150614. doi: 10.1098/rsos.150614 (PMC4736945; doi:10.1098/rsos.150614)
Supplement: Suppl. Table 2. Occurrence of eyespots that surpass the intimidation size threshold discovered in this study. These eyespots have a surface area 28.27 mm2 or larger. [file rsos150614supp8.docx]

**Suppl. Table 2. Occurrence of eyespots that surpass the intimidation size threshold discovered in this study**. These eyespots have a surface area 28.27 mm^2^ or larger.

| Surface Area | Wing sector | Surface | Species | Tribe | Subfamily |
| --- | --- | --- | --- | --- | --- |
| 30.53 | M3 | Ventral anterior | *Morpho helenor* | Morphini | Satyrinae |
| 33.13 | Cu1 | Ventral posterior |  |  |  |
| 39.43 | M3 | Ventral posterior |  |  |  |
| 51.18 | Rs | Ventral posterior |  |  |  |
| 39.22 | Rs | Ventral posterior | *Dynastor darius* | Brassolini | Satyrinae |
| 55.08 | Cu1 | Ventral posterior |  |  |  |
| 42.53 | M1 | Ventral anterior | *Catoblepia orgetorix* | Brassolini | Satyrinae |
| 256.91 | Cu1 | Ventral posterior |  |  |  |
| 35.51 | Rs | Ventral posterior | *Opsiphanes quiteria* | Brassolini | Satyrinae |
| 203.22 | Cu1 | Ventral posterior | *Caligo telamonius* | Brassolini | Satyrinae |
| 66.42 | Cu1 | Ventral posterior | *Opoptera syme* | Brassolini | Satyrinae |
| 39.70 | M1 | Dorsal posterior | *Zethera incerta* | Zetherini | Satyrinae |
| 29.63 | Cu1 | Ventral anterior |  |  |  |
| 28.97 | M1 | Ventral posterior |  |  |  |
| 89.95 | M1 | Dorsal anterior | *Neorina crishna* | Zetherini | Satyrinae |
| 39.95 | M1 | Ventral anterior |  |  |  |
| 38.38 | Rs | Ventral posterior |  |  |  |
| 65.09 | Rs | Ventral posterior | *Taenaris cyclops* | Amathusini | Satyrinae |
| 33.81 | Cu1 | Ventral anterior | *Stichophthalma howqua* | Amathusini | Satyrinae |
| 49.41 | Cu1 | Ventral posterior |  |  |  |
| 84.80 | Cu1 | Ventral posterior | *Thauria aliris* | Amathusini | Satyrinae |
| 45.88 | Cu1 | Ventral posterior | *Amathusia phidippus* | Amathusini | Satyrinae |
| 52.09 | Rs | Ventral posterior | *Amathuxidia amythaon* | Amathusini | Satyrinae |
| 29.22 | Cu1 | Dorsal posterior | *Tisiphone abeona* | Satyrini | Satyrinae |
| 34.21 | Cu1 | Ventral posterior |  |  |  |
