# Supplementary material for: Attack risk for butterflies changes with eyespot number and size
Source: R Soc Open Sci. 2016 Jan 20;3(1):150614. doi: 10.1098/rsos.150614 (PMC4736945; doi:10.1098/rsos.150614)
Supplement: Suppl. Table 3: Eyespot size for species in which at least one eyespot has a surface area 28.27 mm2 or larger. For each species we measured eyespot surface area in up to 18 different wing sectors (Suppl. Fig. 3). The 18 wing sectors selected for measurement were those that contained the largest eyes [file rsos150614supp9.docx]

**Supplementary Table 3:** Eyespot size for species in which at least one eyespot has a surface area 28.27 mm^2^ or larger. For each species we measured eyespot surface area in up to 18 different wing sectors (Suppl. Fig. 3). The 18 wing sectors selected for measurement were those that contained the largest eyespot in at least one of the 255 species examined.

|  |  | | | **Dorsal** | | | | | | | **Ventral** | | | | | | | | | | | |
| --- | --- | --- | --- | --- | --- | --- | --- | --- | --- | --- | --- | --- | --- | --- | --- | --- | --- | --- | --- | --- | --- | --- |
|  |  | | | **Anterior** | | | | **Posterior** | | | **Anterior** | | | | | | **Posterior** | | | | | |
| **Tribe** | **Species** | **M1** | **M2** | **M3** | **Cu1** | **M1** | **Cu1** | | **R5** | **M1** | | **M2** | **M3** | **Cu1** | **Rs** | **M1** | | **M2** | **M3** | **Cu1** | **Pc** |  |
| Morphini | *Morpho helenor* |  |  |  |  |  |  | |  | 19.1 | |  | 30.5 | 21.2 | 51.2 |  | |  | 39.4 | 33.1 |  |  |
| Brassolini | *Dynastor darius* |  |  |  |  |  |  | |  |  | |  |  |  | 39.2 |  | |  |  | 55.1 |  |  |
|  | *Catoblepia orgetorix* |  |  |  |  |  |  | |  | 42.5 | |  |  |  |  |  | |  |  | 256.9 |  |  |
|  | *Opsiphanes quiteria* |  |  |  |  |  |  | |  | 14.9 | |  |  |  | 35.5 |  | | 4.2 |  | 14.7 |  |  |
|  | *Caligo telamonius* |  |  |  |  |  |  | |  | 7.6 | |  |  |  |  | 7.6 | |  |  | 203.2 |  |  |
|  | *Opoptera syme* |  |  |  |  |  |  | |  | 6.9 | |  |  |  | 35.0 |  | |  |  | 66.4 |  |  |
| Zetherini | *Zethera incerta* |  |  |  | 21.0 | 39.7 | 21.2 | |  |  | |  |  | 29.6 | 16.6 | 29.0 | |  |  | 24.2 | 6.0 |  |
|  | *Neorina crishna* | 90.0 | | 8.7 | 7.1 |  | 14.6 | |  | 40.0 | |  |  |  | 38.4 | 3.1 | | 0.9 | 3.4 | 24.5 |  |  |
| Amathusini | *Taenaris cyclops* |  |  |  |  |  |  | |  |  | |  |  |  | 65.1 |  | |  |  | 10.7 |  |  |
|  | *Stichophthalma howqua* |  |  |  |  |  |  | | 13.4 | 19.3 | | 17.9 | 26.3 | 33.8 | 32.1 | 19.6 | | 21.6 | 29.3 | 49.4 |  |  |
|  | *Thauria aliris* |  |  |  |  |  |  | |  |  | |  |  |  | 38.4 |  | |  |  | 84.8 |  |  |
|  | *Amathusia phidippus* |  |  |  |  |  |  | |  |  | |  |  |  | 32.3 |  | |  |  | 45.9 |  |  |
|  | *Amathuxidia amythaon* |  |  |  |  |  |  | |  |  | |  |  |  | 52.1 |  | |  |  | 35.7 |  |  |
| Hypocistina | *Tisiphone abeona* | 3.1 |  |  | 11.6 |  | 29.2 | |  | 5.6 | |  |  | 17.6 | 11.3 | 3.9 | |  |  | 34.2 |  |  |
